# Synergistic Effect between Maternal Infection and Adolescent Cannabinoid Exposure on Serotonin 5HT_1A_ Receptor Binding in the Hippocampus: Testing the “Two Hit” Hypothesis for the Development of Schizophrenia

**DOI:** 10.5402/2012/451865

**Published:** 2012-06-07

**Authors:** Victoria S. Dalton, Mathieu Verdurand, Adam Walker, Deborah M. Hodgson, Katerina Zavitsanou

**Affiliations:** ^1^Schizophrenia Research Institute, Sydney, NSW 2010, Australia; ^2^Department of Psychiatry and Trinity College Institute of Neuroscience, Trinity College Dublin, Dublin, Ireland; ^3^Australian Nuclear Science and Technology Organisation, Lucas Heights, NSW 2234, Australia; ^4^Laboratory of Neuroimmunology, School of Psychology, The University of Newcastle Australia, Newcastle, NSW 2300, Australia; ^5^School of Psychiatry, Faculty of Medicine, University of New South Wales, Sydney, NSW 2052, Australia; ^6^Schizophrenia Research Laboratory, Neuroscience Research Australia, Randwick, NSW 2031, Australia

## Abstract

Infections during pregnancy and adolescent cannabis use have both been identified as environmental risk factors for schizophrenia. We combined these factors in an animal model and looked at their effects, alone and in combination, on serotonin 5HT_1A_ receptor binding (5HT_1A_R) binding longitudinally from late adolescence to adulthood. Pregnant rats were exposed to the viral mimic poly I:C on embryonic day 15. Adolescent offspring received daily injections of the cannabinoid HU210 for 14 days starting on postnatal day (PND) 35. Hippocampal and cortical 5HT_1A_R binding was quantified autoradiographically using [^3^H]8-OH-DPAT, in late adolescent (PND 55), young adult (PND 65) and adult (PND 90) rats. Descendants of poly I:C treated rats showed significant increases of 15–18% in 5HT_1A_R in the hippocampus (CA1) compared to controls at all developmental ages. Offspring of poly I:C treated rats exposed to HU210 during adolescence exhibited even greater elevations in 5HT_1A_R (with increases of 44, 29, and 39% at PNDs 55, 65, and 90). No effect of HU210 alone was observed. Our results suggest a synergistic effect of prenatal infection and adolescent cannabinoid exposure on the integrity of the serotoninergic system in the hippocampus that may provide the neurochemical substrate for abnormal hippocampal-related functions relevant to schizophrenia.

## 1. Introduction

Schizophrenia is a chronic, severe and disabling brain disease, affecting approximately one percent of the population worldwide [1]. The overt signs and symptoms of schizophrenia do not usually manifest until late adolescence but it is believed that the disorder arises from genetic and/or environmental factors encountered prior to disease onset. Amongst the early insults that increase the risk for schizophrenia, prenatal exposure to viral or bacterial infection has been implicated as a strong risk factor by epidemiological studies [2]. The mechanisms by which brain development is altered in offspring following maternal infection remain unknown but it appears that maternal immune activation and in particular proinflammatory cytokines produced in order to fight the infection, rather the infection per se, is responsible [3–5]. Although late risk factors have been difficult to identify, various lines of evidence suggest an association between cannabis use in adolescence and the onset of schizophrenia symptoms. Cannabis increases the risk for psychotic outcomes in a dose-response manner [6]. However, only a minority of cannabis users develop psychosis suggesting that cannabis may interact with other genetic and/or environmental susceptibilities in order to precipitate the disorder (“two hit” hypothesis).

Besides its involvement in depressive illness, serotonin (5HT) insufficiency has been suggested to play an important role in the pathogenesis of the negative symptoms of schizophrenia [7]. The serotonin 5HT_1A_ receptor (5HT_1A_R) has been implicated in both the pathophysiology and pharmacotherapy of schizophrenia [7]. Postmortem studies have reported increased 5HT_1A_R density in several brain regions [8–10]. Recent pharmacological advances indicate that the 5HT_1A_R may be a promising target for alleviating antipsychotic-induced extrapyramidal symptoms and cognitive/affective (e.g., anxiety and depression) disorders in schizophrenia [11].

In animal studies, decreased 5HT levels have been reported in several brain regions of offspring following perinatal exposure to agents that induce immune system activation, for example, the viral and bacterial mimics polyriboinosinic-polyribocytidilic acid (poly I:C) and lipopolysaccharide (LPS) [5, 12, 13]. Decreased 5HT turnover in the hippocampus of mice has been observed after prenatal poly I:C treatment [14]. Treatment with risperidone, which possesses serotonergic antagonistic properties, during the prodromal-like period can prevent the emergence of abnormal behaviours and associated structural brain pathology in the hippocampus of rats exposed to poly I:C in utero [15]. Furthermore, administration of fluoxetine, a serotonin reuptake inhibitor, attenuates prepulse inhibition deficits and enhances amphetamine sensitivity in the poly I:C mouse model [16]. Alterations in the development of the serotoninergic system may, therefore, represent a means by which maternal immune activation causes pathological changes in brain development and behaviour in offspring relevant to schizophrenia.

Animal studies suggest that cannabinoids also interact with 5HT mechanisms. Cannabinoid CB_1_ receptor (CB_1_R) agonists suppress electrically and calcium-stimulated 5HT release from cortical slices [17] and in the hippocampus, delta 9 tetrahydrocannabinol (THC) has been shown to inhibit 5HT release [18]. Conversely, CB_1_R antagonists stimulate 5HT release in the medial prefrontal cortex [19]. Other studies suggest that administration of cannabinoids impairs 5HT-mediated behaviours in laboratory animals [20]. We have previously shown that treatment with the synthetic cannabinoid HU210 increased 5HT_1A_R binding (measured 24 hours after the last injection) in the brain of adult but not adolescent rats suggesting different adaptive responses in the two age groups [21].

In the present study, we examined the impact of prenatal immune challenge by the viral mimic poly I:C (first hit) alone and in combination with adolescent HU210 exposure (“two hit” model) on 5HT_1A_R binding in the cortex and hippocampus of late adolescent, young adult and adult rats. We hypothesised that maternal immune activation will alter serotonergic binding in the developing rat brain and that these alterations may also be affected by adolescent cannabinoid exposure. We found that poly I:C treatment alone increased 5HT_1A_R binding in the hippocampus at all developmental points, an effect that was strengthened by cannabinoid treatment in the “two hit” model.

## 2. Materials and Methods

### 2.1. Animals

Pregnant Wistar rat dams were sourced from the Animal Resource Centre, Perth, Australia. The animals were kept at a constant temperature of 22 ± 2°C on a 12–12 h light-dark cycle with lights on at 09.00 am and were handled during the seven days preceding poly I:C treatment. All handling of animals and procedures were approved by the Animal Care and Ethics Committee at the Australian Nuclear Science and Technology Organisation.

### 2.2. Prenatal Poly I:C Treatment

**Figure 1:**
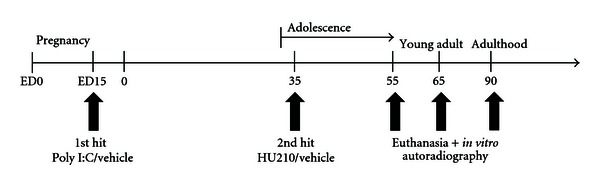
Experimental design. Vertical arrows indicate time-points of administration of poly I:C on embryonic day (ED) 15 to pregnant dams (first hit), administration of the cannabinoid, HU210, to offspring for 14 days beginning on postnatal day 35 (second hit), and euthanasia of offspring on postnatal day 55, 65, or 90.

On embryonic day (ED) 15, pregnant rat dams were placed in a restraint device and received either a single intravenous (i.v.) injection of 4 mg/kg poly I:C (purchased from Sigma, Australia) dissolved in phosphate buffered saline (PBS, *n* = 4) or an equivalent volume of PBS (*n* = 4). Treatment of rats and experimental design is summarised in Figure 1. Injections were administered at a volume of 1 mL/kg. Blood was collected from the tail vein 2 hours postinjection into EDTA (Ethylenediaminetetraacetic acid) coated microtainers (purchased from Becton Dickinson). Rats were then weighed in the four days following the i.v. injections.

Litter sizes ranged from 4 to 14 pups. On PND 21, pups were weaned and dams were euthanized. Blood was again collected from dams at the point of euthanasia.

### 2.3. Adolescent HU210 Treatment of Offspring

On PND 27, male offspring were divided into the following 4 treatment groups: (1) vehicle only: prenatal PBS with adolescent vehicle exposure (VEH-VEH), (2) adolescent HU210 only: prenatal PBS (Phosphate buffered saline) with adolescent HU210 (VEH-HU210), (3) poly I:C only: prenatal poly I:C with adolescent vehicle (POLY-VEH), (4) “two hit” group of prenatal poly I:C with adolescent HU210 (POLY-HU210), see Figure 1. They were housed 3-4 per cage and were handled during the seven days preceding HU210/vehicle treatment.

Treatment of adolescent rats began on PND 35. The synthetic cannabinoid, HU210 (Sapphire Laboratories, Australia), was dissolved in a vehicle solution of Tween 80 : dimethyl sulfoxide : saline (1 : 1 : 98). Rats in the adolescent HU210 groups (VEH-HU210 and POLY-HU210) received daily intraperitoneal injections of 100 *μ*g/kg HU210 for 14 days. Rats in the adolescent vehicle groups (VEH-VEH and POLY-VEH) received vehicle solution for the treatment period. Injections were administered at a volume of 1 mL/kg.

Animals were euthanized on PNDs 55, 65, and 90 (*n* = 6–8 per treatment group at each time point) corresponding to late adolescence, early adulthood and adulthood, respectively, in rats [22, 23]. Blood was collected at the point of euthanasia on PND 55 and 65. Brains were removed and frozen in liquid nitrogen. Coronal brain sections (16 *μ*m) were cut with a cryostat and thaw mounted onto microscope slides.

### 2.4. Analysis of Plasma Cytokine and Corticosterone Levels

Following collection, blood was centrifuged for 20 min at 1000 g at 4°C. Plasma was collected and frozen at −80°C until assayed. Plasma TNF-*α* and IL-6 concentrations were assessed using a Quantikine Rat IL-6, and Quantikine Rat TNF-*α* Immunoassay kit, respectively (R&D Systems, USA). Enzyme-linked immunosorbent assays (ELISA) were performed according to kit instructions. The mean recovery of TNF-*α* in plasma is reported to be 96% with a mean inter- and intra-assay variability of 9.36% and 3.13%. The mean recovery of IL-6 in plasma is reported to be 96% with a mean inter- and intra-assay variability of 9.31% and 3.11%, respectively. Plasma corticosterone concentrations were assessed using a rat corticosterone ^125^I radioimmunoassay kit (MP Biomedicals, USA). The recovery of exogenous corticosterone is 100%, with a mean inter- and intra-assay variability of 4.4% and 6.5%, respectively.

### 2.5. Autoradiography

Binding for 5HT_1A_R was carried using [^3^H]8-OH-DPAT. Sections were preincubated for 15 min at room temperature in a buffer containing 50 mM Tris HCl (pH 7.4), 120 mM NaCl and 4 mM CaCl_2_. Sections were then incubated for 60 min at room temperature in the same buffer with the addition of 2 nM [^3^H]8-OH-DPAT (specific activity 170.2 Ci/mmol, Perkin Elmer, USA). Nonspecific binding was determined by incubating adjacent sections in 2 nM [^3^H]8-OH-DPAT in the presence of 10 *μ*M MM 77 dihydrochloride. After this incubation, sections were washed twice for 10 min each in ice cold buffer, followed by one dip in ice-cold distilled water and then dried. Sections were opposed to Kodak Biomax MR films, together with autoradiographic standards ([^3^H] microscales from Amersham), in X-ray film cassettes. Films were exposed for 49 days and were then developed and fixed using Kodak GBX developer and fixer.

### 2.6. Quantitative Analysis of Autoradiographic Images

Films were analyzed by using a computer-assisted image analysis system, Multi-Analyst, connected to a GS-690 Imaging Densitometer (Bio-Rad, USA). Quantification of binding was performed in the CA1 region of the hippocampus and in the primary somatosensory cortex, layers I–III (CTXUP) and layers IV–VI (CTXDO), by measuring the average optical density in adjacent sections. Nonspecific binding was subtracted from the total binding to determine the specific binding. Optical density measurements for specific binding were then converted into fmoles [^3^H]8-OH-DPAT per mg tissue equivalent (fmol/mg TE), according to the calibration curve obtained from the tritiated standards.

### 2.7. Statistical Analysis

The effect of treatment on body weight over time was analyzed in pregnant dams using two-way ANOVA (body weight × day) with repeated measures and Bonferroni's post hoc tests. Cytokines and corticosterone levels in poly I:C treated pregnant rat dams were compared with controls using a Student's *t*-test. Cytokines and corticosterone levels were compared in offspring using one-way ANOVA with LSD post hoc tests. Two-way ANOVA with treatment and brain region as independent variables, followed by post hoc Bonferroni tests, was conducted on each postnatal time point in order to identify overall statistically significant variations in binding in hippocampus CA1 and cortical subregions assessed on PNDs 55, 65, and 90 across all treatment groups. Data was analysed using GraphPad Prism (CA, USA) and PASW Statistics 18 (IL, USA) statistical packages.

## 3. Results

### 3.1. Effect of Poly I:C Treatment on Body Weight in Pregnant Dams

**Figure 2:**
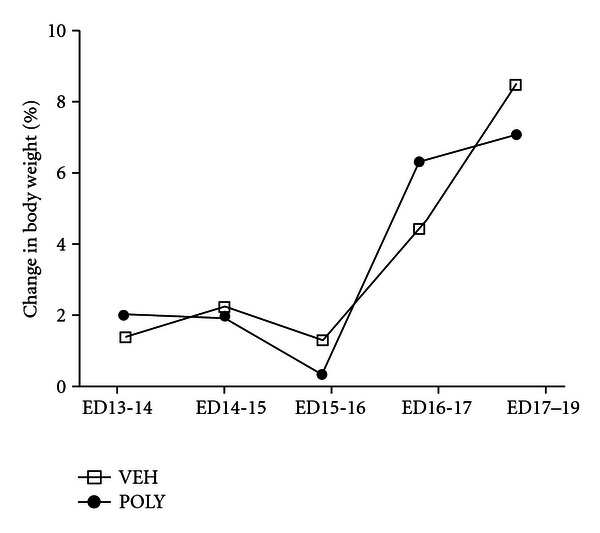
Effect of poly I:C treatment on body weight in pregnant dams. The percentage change in body weight between the embryonic days (ED) indicated is shown for poly I:C (POLY) and vehicle (VEH) treated dams (*n* = 4 per group).

A significant effect of day (*F*(3,18) = 59.53, *P* < 0.0001) but not treatment (*F*(1,18) = 0.4881, *P* = 0.5109) was observed when weight gain was compared over the four days posttreatment. Directly after injection, however, poly I:C treated dams gained an average of 0.3% body weight compared to vehicle-treated animals that gained 1.2% between ED 15 and 16 (Figure 2).

### 3.2. Plasma Cytokines and Corticosterone Levels in Pregnant Dams

**Figure 3:**
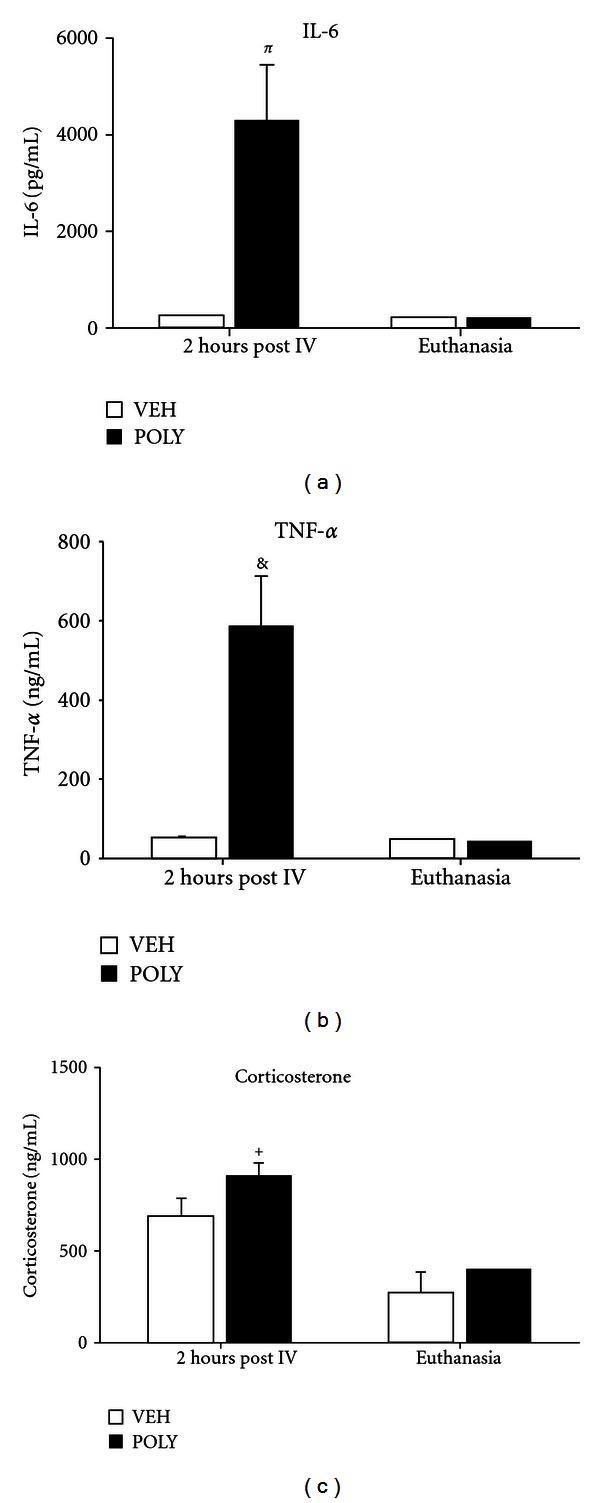
Plasma cytokine (a and b) and corticosterone (c) levels in pregnant dams 2 hours post intravenous (IV) injection with vehicle (VEH) or poly I:C (POLY), or at the point of euthanasia, 27 days after injection (*n* = 4 per group). ^*π*^ *P* = 0.012, ^&^ *P* = 0.005, ^+^ *P* = 0.05 compared to PBS treated dams using Student's *t*-test.

Poly I:C at the dose of 4 mg/kg i.v. provoked a systemic immune response. Plasma cytokines and corticosterone concentrations were assayed in pregnant rats on ED15, 2 hours after poly I:C treatment and at the point of euthanasia, 27 days posttreatment. Poly I:C injection on ED15 caused statistically significant increases in TNF-*α* (*P* = 0.005), IL-6 (*P* = 0.012) and corticosterone (*P* = 0.05) levels compared to PBS-treated rats (Figure 3). Cytokines and corticosterone levels had returned to baseline in poly I:C treated dams 27 days after injection (Figure 3).

### 3.3. Plasma Cytokine and Corticosterone Levels in Offspring on PND 55 and 65

One-way ANOVA revealed a significant effect of treatment (*F*(3,23) = 4.982, *P* = 0.008) on TNF-*α* levels on PND 55. Poly I:C treated rats had higher levels of TNF-*α* compared to vehicle-treated rats (*P* = 0.02) and adolescent HU210 treated rats (*P* = 0.001). The “two hit” group had also higher TNF-*α* compared to adolescent HU210 group (*P* = 0.047), see Table 1. No significant effects of treatment on TNF-*α* levels were observed on PND 65 (Table 1).

No significant effects of treatment on corticosterone and IL-6 levels were observed on PND 55 or 65.

### 3.4. [^3^H]8-OH-DPAT Binding in Offspring on PNDs 55, 65 and 90

Two-way ANOVA at each developmental age revealed a significant effect of treatment on 5HT_1A_ binding, a significant effect of region and a significant interaction between them on all PNDs 55, 65, and 90 (Table 2).

**Figure 4:**
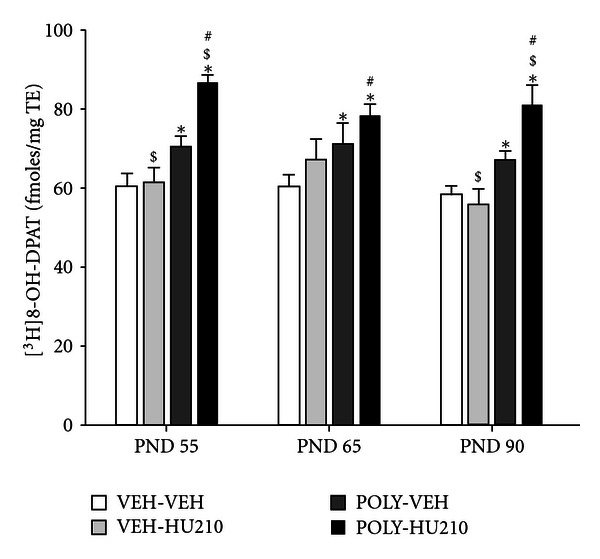
[^3^H]8-OH-DPAT binding (fmoles/mg tissue equivalent (TE)) to serotonin 5HT_1A_ receptor in CA1 region of the hippocampus in offspring from poly I:C treated dams and received 100 *μ*g/kg HU210 (POLY-HU210) or vehicle (POLY-VEH) for 14 days beginning on postnatal day (PND) 35, and offspring from PBS-treated dams and received 100 *μ*g/kg HU210 (VEH-HU210) or vehicle (VEH-VEH). Offspring were euthanized on PNDs 55, 65, and 90. In Bonferroni post hoc tests, 0.0001 < *P < 0.05 compared to VEH-VEH group; 0.0001 < ^$^ *P* < 0.05 compared to POLY-VEH group; 0.0001 < ^#^ *P* < 0.05 compared to VEH-HU210 group (*n* = 6–8 per treatment group at each time point).

**Figure 5:**
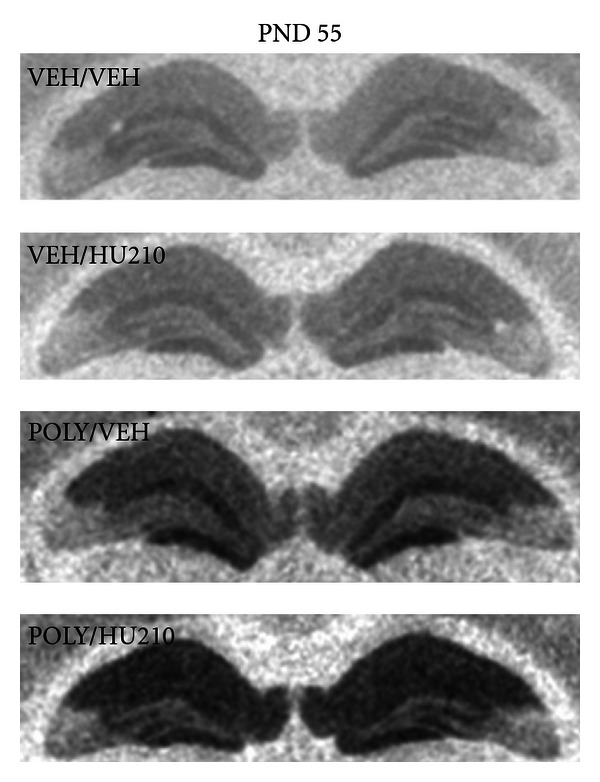
Typical autoradiographs showing [^3^H]8-OH-DPAT binding indicating serotonin 5HT_1A_ receptor density in the CA1 of the hippocampus in offspring euthanized on postnatal day 55.

Post hoc analysis revealed that this significant interaction was due to significant treatment effects in the CA1 region of the hippocampus (Table 3, Figures 4 and 5). More specifically, poly I:C only (first hit) treated offspring showed increases of 15–18% (0.0001 < *P* < 0.05) in binding compared to their respective controls at all developmental ages. Poly I:C treated animals that were also exposed to HU210 during adolescence (“two hit” group) displayed even greater increases in binding in the CA1 compared to controls at all ages (PND 55: 44%, *P* < 0.001; PND 65: 29%, *P* < 0.001; PND 90: 39%, *P* < 0.001). Two hit animals had also higher levels of binding compared to animals exposed to HU210 only at all developmental ages (PND 55: 41%, *P* < 0.001; PND 65: 16%, *P* < 0.05; PND 90: 45%, *P* < 0.001), and higher levels of binding compared to poly I:C only group on PNDs 55 (23%, *P* < 0.001) and 90 (21%, *P* < 0.001). Levels of binding in rats exposed to HU210 alone in adolescence did not significantly differ compared to controls at any time point.

## 4. Discussion

Using a novel two hit model of environment-related psychopathology, the current study demonstrated that maternal immune activation (induced by maternal poly I:C treatment at ED15), alone and in combination with adolescent cannabinoid treatment of offspring, had region-specific and long-lasting effects on 5HT_1A_R binding in the developing rat brain that is, in late adolescent, young adult and adult rats. In particular, an additive effect of poly I:C and HU210 treatment was observed in the hippocampus which resulted in a strong upregulation of 5HT_1A_R binding in the CA1 region of the hippocampus. Importantly, cannabinoid treatment alone during adolescence had no effects on binding at any of the developmental points examined.

 To examine whether poly I:C treatment provoked a systemic immune response, we evaluated the presence of TNF-*α*, IL-6 and corticosterone in the maternal blood 2 hours after poly I:C injection. We observed robust increases in maternal cytokines and corticosterone levels indicative of activation of the maternal immune system. Offspring of poly I:C treated dams also displayed increased levels of plasma TNF-*α* at PND 55 mirroring alterations in cytokines levels reported in people with schizophrenia [24].

 Our results are in line with studies in animals showing that maternal inflammation during gestation leads to anatomical changes in the hippocampus of offspring and alterations in specific behaviours that depend on that structure [15, 25]. Piontkewitz and collaborators [15] have shown, for example, that treatment with risperidone, a serotonergic receptor antagonist, during adolescence prevents the decrease in hippocampal volume caused by prenatal poly I:C in rats. Our results are also in line with postmortem human studies showing elevated 5HT_1A_R binding in the hippocampus in schizophrenia [9] although binding has been shown to be unaffected in another study [26]. A series of human and animal studies suggest that antagonism of 5HT_1A_R provides benefits in treating cognitive impairment in schizophrenia [11]. For example, the new antipsychotic lurasidone combined with a 5HT_1A_R antagonist (weak partial agonist) action [27] has been shown to improve the learning and memory impairment induced by the NMDA receptor antagonist MK801 [28] and to ameliorate the cognitive impairment in schizophrenia patients [29].

Besides its involvement in depressive illness, 5HT insufficiency has been suggested to play an important role on the pathogenesis of the negative symptoms of schizophrenia [7]. One of the emerging mechanisms linking enhanced proinflammatory activity with the induction of affective emotional and social impairment is the central tryptophan metabolism [30]. Tryptophan is an essential amino acid needed for the biosynthesis of 5HT. Enhanced proinflammatory actions in the central nervous system lead to increased tryptophan degradation into kynurenine by indoleamine 2,3-dioxygenase thereby reducing the bioavailability of tryptophan for 5HT synthesis [30]. Consistent with this, decreases in hippocampal levels of 5HT have been reported in mice following prenatal poly I:C and human influenza exposure [5, 12] and in rat offspring after immune system activation induced by LPS treatment [13]. In the current study, poly I:C treatment alone had profound effects on the 5HT_1A_R in the hippocampus causing a 15–17% increase in binding in the CA1 on PND 55, 65, and 90. It is, therefore, tempting to hypothesise that this increase (first hit) reflects an adaptive mechanism to compensate for decreased 5HT levels induced by maternal immune system activation.

Our results suggest that cannabis exposure alone during adolescence is not a sufficient factor to induce developmental changes in the integrity of the 5HT_1A_R system in the hippocampus. Offspring of poly I:C treated rats that were exposed to cannabinoid treatment during adolescence (two hit group) displayed however increases of larger magnitude (29–44%) in hippocampal 5HT_1A_R binding compared to the poly I:C alone group. This finding indicates that cannabis during adolescence can exacerbate neural vulnerabilities which have been laid down early in life, thus enhancing neurochemical outcomes relevant to schizophrenia in adulthood. It is likely that prenatal immune challenge affects the integrity of the serotoninergic system making it vulnerable to subsequent cannabinoid exposure in adolescence. Interestingly, previous studies in adult rodents have shown that, in the hippocampus, THC and the CB1R agonist WIN 55, 212-2 decreased 5HT synthesis [31], and 5HT release was suppressed by THC administration [18]. Based on the above studies it is possible that increased 5HT_1A_R binding in the “two hit” group is a compensatory response for 5HT deficiency induced by prenatal poly I:C exposure that is subsequently exacerbated by cannabinoid treatment in adolescence. Clearly more research is required to test this hypothesis.

The neurochemical alterations observed in both poly I:C and poly I:C/cannabinoid groups in our study lend weight to the “two hit” hypothesis for mental illness, although our study is limited by the absence of functional studies that would be invaluable. Despite this limitation, the current results are presented to further stimulate interest in studies looking at the interactions of early (prenatal) inflammatory effects with late (adolescent) insults (e.g., stress and cannabis) in producing schizophrenia-related functional and/or neurochemical outcomes, an area of research that is a growing field of interest [3, 14, 32]

## 5. Conclusions

Poly I:C treatment at ED15 results in immune system activation and abnormal elevation of 5HT_1A_R binding in the hippocampus of late adolescent, young adult and adult offspring. Adolescent cannabinoid exposure alone is not a sufficient factor to alter the integrity of the 5HT_1A_R system, but when combined with poly I:C leads to greater elevations in binding compared to poly I:C alone. Our results support the biological plausibility for a synergistic effect of prenatal infection and cannabis in adolescence and lead weight to the “two hit” hypothesis for mental illness.

## Figures and Tables

**Table 1:** Effect of prenatal poly I:C exposure and adolescent HU210 treatment on plasma TNF-*α* levels in offspring euthanized on postnatal day 55 and 65 (*n*  = 6-7 per treatment group).

	VEH-VEH		VEH-HU210		POLY I:C-VEH		POLY I:C-HU210	
	PND 55	PND 65	PND 55	PND 65	PND 55	PND 65	PND 55	PND 65
TNF-*α* (ng/mL)	41.3 ± 0.5	60.2 ± 8.8	40.7 ± 0.4	51.5 ± 1.8	43.7 ± 1.0^∗#^	58.1 ± 8.7	41.8 ± 0.5^&^	49.4 ± 0.8

Data are presented as average ± SEM, *P = 0.02 compared to VEH-VEH group; ^#^ *P* = 0.001 and ^&^ *P* = 0.047 compared to VEH-HU210 group in LSD post hoc tests.

**Table 2:** Two-way ANOVA (treatment × region) of serotonin 5HT_1A_ receptor binding in the CA1 of the hippocampus and somatosensory cortex on postnatal days (PND) 55, 65, and 90 (*n* = 6–8 per treatment group at each timepoint).

	Treatment	Region	Treatment × Region
PND 55	*F*(3,63) = 10.03, *P* < 0.0001	*F*(2,63) = 844.3, *P* < 0.0001	*F*(6,63) = 14.24, *P* < 0.0001
PND 65	*F*(3,63) = 3.593, *P* = 0.0183	*F*(2,63) = 529.1, *P* < 0.0001	*F*(6,63) = 3.399, *P* = 0.0057
PND 90	*F*(3,60) = 5.370, *P* = 0.0024	*F*(2,60) = 668.3, *P* < 0.0001	*F*(6,60) = 9.262, *P* < 0.0001

**Table 3:** [^3^H]8-OH-DPAT binding to serotonin 5HT_1A_ receptor in layers I–III (CTXUP) and IV–VI (CTXDO) of somatosensory cortex and CA1 region of hippocampus (CA1).

	VEH-VEH			VEH-HU210			Poly I:C-VEH			Poly I:C-HU210		
	PND 55	PND 65	PND 90	PND 55	PND 65	PND 90	PND 55	PND 65	PND 90	PND 55	PND 65	PND 90
CA1	60.08 ± 3.39	60.37 ± 2.80	58.01 ± 2.23	61.32 ± 3.44	67.16 ± 5.00	55.64 ± 3.97	70.40 ± 2.48	71.19 ± 5.01	66.90 ± 2.04	86.43 ± 1.86	78.09 ± 2.71	80.81 ± 4.6
CTXUP	14.87 ± 1.34	13.40 ± 0.80	12.36 ± 1.18	14.81 ± 0.69	17.21 ± 2.08	11.52 ± 1.25	11.69 ± 0.72	11.50 ± 1.13	10.16 ± 0.98	13.87 ± 1.19	13.02 ± 1.17	9.29 ± 1.22
CTXDO	27.78 ± 1.96	23.72 ± 1.02	25.65 ± 1.56	25.63 ± 1.22	28.16 ± 1.94	23.47 ± 1.25	23.88 ± 2.06	23.41 ± 1.50	22.01 ± 1.29	24.42 ± 1.24	24.96 ± 1.03	23.11 ± 1.51

Data are presented as average ± SEM of *n*Ci/mg Tissue Equivalent (*n* = 6–8 per group).
